# Foreign

**Published:** 1841-06-05

**Authors:** 


### FOREIGN.

*History of the last illness of Sir A. P. Cooper, Bart., and examination of the body after death.* For many months previous to his last illness, Sir Astley Cooper had occasionally experienced great dyspnœa, upon the slightest exertion: and it had been observed by his friends that the peculiarity of his complexion bespoke some serious impediment to the circulation. It was not, however, till about six weeks before his death that he found difficulty in assuming the recumbent posture; and about that time he began to pass the greater part of his nights in the arm-chair, rather than attempt to lie down. He still continued to see a few patients during the day, both at home and at their own houses. He now became the subject of frequent cough; which was immediately brought on, if he attempted to recline. The gout, of which he had for several years experienced periodical attacks, showed itself imperfectly in the fore-finger of the left hand; and his legs began to swell, owing to the depending position in which they constantly remained.

During all this time he refused medical aid; and it was not till the 22d of January that he consented to see any one, to whom he might state his symptoms. At the time he was first visited, he was sitting in his chair, with his body inclined forward, and his chin nearly resting on his chest; the pulse accelerated; not the slightest *bruit* nor abnormal sound in the heart, though the beat was extensive, and heard quite to the right side of the chest. The lungs afforded considerable bronchial rattle, but were neither consolidated nor compressed, and filled both cavities of the chest.

Although remedies appeared more than once to produce a temporary remission of his symptoms, and a further attack of gout in one foot seemed to afford some relief to the chest, yet, upon the whole, the disease advanced, and was attended by frightful fits of dyspnœa, during which his face was purple and his mind confused; and it was in one of these paroxysms that he died, on the morning of the 12th of February.

Shortly before his death, Sir Astley Cooper expressed a wish that the appearances which should be presented on the inspection of his body might be recorded in the Guy's Hospital Reports. He had particularly alluded to four points, the investigation of which he thought desirable;—a cured oblique inguinal hernia; a cured umbilical hernia; some suspected indications of phthisis in his youth; and an inability to sleep whilst lying on his left side.

*Examination of the body of Sir Astley P. Cooper, Bart., in the 73d year of his age, on February 13th,* 1841, *at* 9 *o'clock in the evening,* 32 *hours after death, by Mr. John Hilton, in the presence of Dr. Chambers, Dr. Bright, Mr. C. A. Key, and Mr. Edward Cock.*

The weather was warm and damp: there were slight cadaverous indications, from gravitation towards the posterior part of the corpse: the face and anterior surface of the body exsanguine: there was a general and extensive œdema of the lower extremities; but no evidence of serous infiltration in the arms, nor in any other part of the surface of the body.

The head was not examined.

A globular projection, about the size of a large nut, was found at the umbilicus; which receded on pressure, leaving a well defined rounded aperture in the linea alba, capable of admitting the end of the little finger. This protrusion consisted of a few congregated lobes of fat placed immediately behind the umbilicus, between it and the peritoneum, the free surface of which was corrugated, and presented a puckered appearance, most probably inflammatory, and the result of the artificial curative means which had been employed for a long period during life.*

The anterior, thoracic, and abdominal parietes were covered with a layer of fat, about an inch in thickness, soft, and oleaginous. The muscular tissue exposed during the inspection was pale, soft, and flabby: indeed, the latter expression is applicable to nearly all the tissues. No gaseous or fluid effusion was found in the cavity of the peritoneum: the greater omentum, loaded with adipose matter, was contracted, and did not extend downwards more than two inches from the transverse colon. Some very old membranous adhesions existed between the right angle of the colon and the gall-bladder: cadaveric transudation of the bile from this viscus had slightly tinged the surrounding parts.

The viscera occupied their natural positions; excepting the cœcum, which was completely invested by the peritoneum, and hence less fixed than usual.

The liver healthy in form: some parts of its surface were slightly contracted and uneven; and sections of it presented hepatic venous congestion, approaching what is termed a "nutmeg appearance."

The gall-bladder was small; and contained a moderate quantity of healthy bile, which, upon gentle pressure, passed rapidly into the duodenum.

The spleen was rather larger than natural, its capsule a little opaque, and the interior of the organ very firm; a section presenting a smooth solid surface of a purplish gray colour.

The stomach was large, and distended with gas; the cardiac extremity stained brown by cadaveric transudation, or the action of the gastric fluid upon the blood: its tissues appeared quite healthy.

The small intestines presented nothing abnormal: nor was there any thing remarkable in the large intestines, excepting the dilated condition of the cœcum, the parietes of which were thin; its mucous membrane congested.

The pancreas was healthy.

The kidneys were surrounded by a considerable quantity of adipose tissue, remarkably dense, and very firmly adherent to the fibrous capsule of the gland. Both kidneys were much congested with blood, rather larger than

* Sir Astley Cooper wore a piece of cork adapted to the umbilicus; and maintained in its place by straps of adhesive plaster, during many years, and until his fatal illness.

natural, their surfaces mottled, and slightly granular. These morbid conditions were the most evident at the lower part of the left kidney; less advanced but more generally diffused, in the right: and on the anterior surface of the latter, near its convex edge, were found two small cysts, containing a straw-coloured fluid.

The supra-renal capsules were healthy.

The urinary bladder was healthy and contracted, and contained about two drachms of whitish turbid urine.

The internal abdominal ring, on the left side, was rendered distinct by a tubular extension of the peritoneum for about an inch into the inguinal canal.

A depression existed in a corresponding situation on the right side, the bottom of which was firm, irregular, and corrugated; and upon very careful examination, a minute serous canal, not more than a line in breadth when opened, was traced extending from it, along the spermatic cord, into the cavity of the tunica vaginalis, being the remains of a congenital inguinal hernia.*

Upon raising the sternum and cartilages of the ribs, both lungs were brought into view; and retained their expanded condition, overlapping the pericardium, and manifesting no disposition to collapse. No pleuritic adhesions existed on either side of the chest; nor was there any effusion, except into the right pleural cavity, which contained about three ounces of sanguinolent rather turbid serum.

A little recent pleuritis was found on the middle lobe of the right lung, rendering it slightly adherent by plastic effusion to the adjoining limbs to a small extent. Both lungs presented general vesicular emphysema to a very great degree, and their edges were more rounded than natural.

The larynx was not examined.

The lining membrane of the trachea and larger bronchi was smooth, but of a dark purple hue, from congestion in the minute blood-vessels: the same appearances extended throughout the bronchial ramifications, the smaller of which were filled with a very tenacious puriform mucus; and many of them were observed much dilated. Both lungs were extremely congested with dark blood, especially in and near the central portions of their lobes. At the superior and posterior part of the right lung was a small depressed and somewhat contracted surface, about the extent of a sixpence; a section of which exposed a calcerous mass, very uneven upon its surface, and about equal to the size of a small pea: it was placed about three lines distant from the pleura.

When the pericardium was opened, the heart was seen, very large and distended; and about two ounces of rather dark or brown-coloured slightly turbid serum occupied the posterior part of the cavity.

The right auricle and ventricle filled with very dark-coloured imperfectly-coagulated blood. The auriculo-ventricular valves sound. Through one of the pulmonary valves, near its angle of union with an adjoining valve, was a perforation nearly the size of a small goose-quill. A tolerably firm fibrinous coagulum was found in the pulmonary artery and its branches, extending, by minute prolongations, to the fifth divisions; these were made evident, by withdrawing them in a continuous mass with the forceps.

The left auricle and ventricle were occupied by a large quantity of black grumous half-liquid blood. A large portion of the mitral valve opaque, and a little thickened; otherwise healthy. The aortic valves thickened, and rather rigid at their attached margins: whilst the free margins presented a remarkably healthy appearance for their age.

The left ventricle was much dilated; its apex much broader, and more prolonged than natural: the parietes somewhat hypertrophied; and the muscular fibres of the whole organ were pale, flabby, and weak.

The aorta, which was small and narrow, pursued its usual course, but gave off the left vertebral artery between the left common carotid and left subclavian. The entrance to the arteria innominata was contracted, and slightly irregular.

Many small irregular yellowish opaque patches were seen under the lining membrane of the thoracic aorta and the ascending portion of the left subclavian artery. In most of the parts so affected, the internal membrane was much softened, breaking down under slight pressure: at three or four points it was destroyed to a small extent, admitting a thin layer of dark matter, probably altered blood, separating it in a slight degree from the subjacent tissue: this latter state was noticed near the origin of the arteria innominata and the commencement of the descending aorta. The whole length of the abdominal aorta was full of black grumous blood; its parietes thickened; the lining membrane opaque, and raised by the sub-deposition of hard, almost bony matter.—*From Guy's Hospital Reports, in London Med. Gazette.*

---

*On the History of the Employment of Cinchona Bark in the treatment of Acute Rheumatism.* By DAVID D. DAVIS, M. D., Professor of Midwifery in University College, London.—On perusal of my late communication on acute rheumatism, it possibly occurred to the reader that I considered the merit and originality of prescribing bark as a remedy for that malady as due to the late Dr. Haygarth, of Bath, who had, indeed, previously resided at Ches-

* Sir Astley Cooper wore a truss on the right inguinal canal, from the age of nineteen to twenty-five.

ter for several years. A statement corrective of such an inference seems necessary to do justice to the almost unequalled candour and modest unpretentious merit of that excellent writer. As the practice may appear altogether a modern novelty to the greater part of the profession of the present day, a brief account of its adoption by Dr. Haygarth, and of the singular manner in which he became himself acquainted with it, cannot fail to prove interesting to many readers of the Lancet. Dr. Haygarth shall give that account in his own words: "For several years after the period when I commenced the practice of physic at Chester, that excellent physician, the late Dr. John Fothergill, used annually to retire from the fatigues of his profession during about two months in the summer to Lea-Hall, in Cheshire. In this pleasing rural retreat, I had frequently opportunities of enjoying his very improving and entertaining conversation. He allowed me the very important privilege of stating to him the doubts and difficulties which often perplexed me as a young physician.

"With a truly liberal and enlightened mind, he frequently communicated to me his opinion and advice whenever he was thus consulted.— In one of these friendly visits, I solicited his counsel for a patient ill of rheumatic fever. He recommended that the Peruvian bark should be administered. At this advice I expressed great surprise, observing that it was directly contrary to the mode of treatment which I had been taught by the most judicious and learned authors and professors, and that I had always understood the bark to be highly improper in all inflammatory disorders. To my objections he replied,—'When I was a young physician, in consequence of being twice called out of my bed to visit patients on a frosty night, I caught a very severe rheumatic fever. By the advice of my medical brethren, I had been blooded repeatedly, and largely, even to seventy ounces. My disease remained unsubdued, and my blood still exhibited an inflammatory crust; hence I was convinced that the method of curing this fever by such copious evacuations was erroneous: soon after my recovery I was desired to visit a patient lli of an acute rheumatism.

"'At my request, Sir Edward Hulse, at that time the most eminent physician in London, was consulted. He proposed that we should order the Peruvian bark. I gladly agreed to the proposal, as I thought there were several analogies between an ague and a rheumatic fever; in both diseases the urine lets fall a similar lateritious sediment; in intermittent, as well as rheumatic fevers, the blood, when drawn, is covered with an inflammatory crust; the pain and fever of rheumatism have certain periodical, although not quite regular paroxyms, and intermissions. In this consultation with Sir Edward Hulse, the bark was given with such manifest advantage, that I haev ever since adopted the practice, and I recommend it to you in spite of all medical authorities to the contrary.'"

To inquire into the origin and practice of giving bark in acute rheumatism, would be curious and instructive. In Dr. Richard Morton's "Treatise on Fevers," that learned author describes the proteiform nature of agues. After explaining that the poison of intermittent fevers was often the cause of hemicrania and apoplexy, he says, "I have a hundred times observed that the colic of the stomach and of the intestines—that the acutest spasmodic pleurisy —that general and local rheumatism—that scarlet and erysipelatous fevers, with the strongest pathognomonic symptoms, were produced by this poison; these symptoms returned at stated periods; the urine was like what is voided in intermittents; and these fevers, either spontaneously or by remedies, drop their mask, when I cure them soon, constantly and happily, by cinchona."

The accuracy of a part of this reasoning may perhaps be questionable; nor does the whole of it necessarily belong to our present subject. An useful discovery may sometimes result from an erroneous theory. In illustration of this point, Dr. Haygarth appositely enough quotes the following case, from Morton's work already referred to, vol. i., p. 249 :—

"A dyer, in Whitecross street, was attacked with an almost universal rheumatic pain, wandering through all his limbs. After he had been frequently thrown into a syncope, and his life had been despaired of from the violence of his spasmodic pains, I was at length consulted. When I had observed that his urine was of a deep red colour, and upon exposure to the air that it deposited a lateritious sediment, and was informed by his attendants that his pains increased at stated periods every day, or every other day, and that the exacerbations were accompanied with the greatest anxiety, I announced that these painful spasms originated from the poison of intermittent fevers; wherefore I ordered twelve ounces of blood to be taken from the arm to relieve the present pain; and, after an interval of six hours, I ordered an antimonial emetic. By these remedies the spasms were soon relieved; but that I might perfectly cure the disorder, I ordered a drachm of bark, with a few drops of laudanum, to be given every third or fourth hour. By these means I intended to destroy the morbific poison, lest the spirits again irritated should excite a new paroxysm. After he had taken an ounce and a half of fresh, good bark, he was immediately relieved, without any other remedy, from the rheumatic fever and spasms. The natural urine and appetite returned, and the patient was restored to health. In a fortnight after, the energy of the bark had begun to fail: he again suffered a relapse of the rheumatism, which, after being bled, was cured with equal facility by the bark, without the aid of any other remedy, as happens to those who are afflicted with a relapse of an intermittent fever." "Thus," observes Dr. Haygarth, "I was much delighted to discover by what traditional authority this practice had been transmitted from one physician to another, as clearly appears from the following history :—In Morton's 'Treatise on Fevers,' and the chapter above quoted, the twentieth case is entitled, 'An Ague, long concealed under the mask of a pain of the breast, which was, in reality, rheumatic.' The symptoms and remedies of this case are so various, that it would be tedious to read them all. In this manner, he adds, by trusting to false principles, I had brought my patient almost to the jaws of death, being worn down by her fever, watchfulness, delirium, and pain, until the fever, coming every day at stated periods, betraying its type and nature, I suspected that the lurking febrile poison was the cause of this most painful symptom. Wherefore, with the consent of my celebrated colleague, Dr. Hulse, who was called into consultation with me on this case, I ordered blood again to be taken from her arm on the 3d of April, 1690, to diminish the violent efforts of the spirits, which I thought were the cause of the pain; and to destroy the febrile poison, twelve drachms of the Peruvian bark, mixed with an equal quantity of white sugar, were divided into doses of two drachms each, to be taken every four hours. A cordial julep was occasionally given. On the fifth day after these remedies had been administered, far beyond the hopes of her friends and my own, I beheld our patient without fever, cheerful, lively, sleeping placidly, nearly quite free from pain, and all other complaints." In this manner, observes Dr. Haygarth, I discovered, very highly to my satisfaction, by what traditional authority, supported by experience, in spite of the powerful influence of a contrary hypothesis, the benefit to be derived from the Peruvian bark, in acute rheumatism, had been preserved from oblivion by three physicians of uncommon abilities,—Morton, Hulse, and Fothergill. It may not be improper to remark, that this tradition seems to have had, as might be expected, more influence in London than in any other place. Sir John Pringle, in his observations on the Diseases of the Army, p. 166, says, some physicians have ventured to give the bark in acute rheumatism after plentiful bleeding, and as soon as a sediment appeared in the water, although some degree of fever might remain, and the pains might still be considerable. I have had some success myself in giving it thus early, but I have not seen a sufficient number of cases to enable me to recommend the practice to others.

The first cases of rheumatic fever in which Dr. Haygarth prescribed the Peruvian bark, on the recommendation of Dr. Fothergill, occurred in 1769. The favourable opinion which I entertained of this singular practice, received on such respectable authority, was soon confirmed by my own experience of its efficacy. At the date of publication of Dr. Haygarth's "Clinical History of Acute Rheumatism," he observes, that during the long period which had elapsed since his adoption of the practice, amounting to five-and-thirty years, he had never lost an opportunity of prescribing the Peruvian bark, which appeared to him proper for its use; but always, at first, with great caution, and after sufficient evacuations from the blood-vessels, stomach, bowels, and the skin. Taught by attentive observation and successful experience, he gradually employed this great remedy with more and more freedom, and it was attended with still more manifest proofs of its safety and efficacy. Dr. Haygarth exhibited the bark in all cases of acute rheumatism. In thirty-five cases out of sixty-six, the bark was given during the first fortnight of the disease, from the first to the fifteenth day inclusive of the fever; in eighteen cases, during the next month, being from the sixteenth to the fortieth day of the disease. As to the remaining eleven cases, they may rather be reckoned complaints, in consequence of acute rheumatism, than examples of the disease itself. Out of eighty-four cases, which we find recorded in Dr. Haygarth's tables, the bark was ordered on the first day of the physician's visit; although this must not be too rigidly construed, as cases now and then occurred where the exhibition of the remedy was delayed until the next, or some early subsequent day. In a few of the cases, the bark was ordered at the commencement, without any preparatory measures, although in the great majority of cases a sufficient number of evacuations had been previously obtained by antimony. The form and dose in which the Peruvian bark was exhibited, are fully explained in Dr. Haygarth's first table, whence it appears that the powder was given in eighty-two cases, the decoction in thirty, and the tincture in nine cases.

The powder was exhibited in doses of between five and sixty grains, and the repetition of the quantity prescribed was ordered, from once in two to once in twelve hours. But the most common dose was from ten to thirty grains, and the usual time of repeating it was from the third to the eighth hour. The decoction of bark was given, from between the dose of an ounce, and an ounce and a half, up to two ounces every second, fourth, sixth, or eighth hour. The tincture of bark was ordered in nine cases; but, I believe, never until both the fever and inflammation were gone, or much abated.

Physicians have observed that acute rheumatism is scarcely ever a fatal disease. This observation may be true, and is confirmed by my own experience; whilst it remains in its proper seat, the muscles and joints, and when not combined with other mortal maladies; but out of one hundred and seventy cases which occurred in the practice of Dr. Haygarth, during a period of five-and-thirty years, he met with twelve which had a fatal termination, either by a translation of the inflammation to the brain, lungs, kidneys, stomach, or some other vital part, has been found in combination with other diseases. In the second section of Dr. Haygarth's "Clinical History," consisting principally of illustrative matter, a full statement of all the unfavorable cases is especially detailed. As the principal purpose of the history was to ascertain how far the Peruvian bark should be considered a safe and salutary remedy in acute rheumatism, the author showed the utmost solicitude to inquire, with all possible accuracy, how far the fatal cases related in the second section should excite any doubt relative to this question. Of the twelve patients who actually died of the disease, it is a fact of obvious importance, that only four died of acute rheumatism. Moreover, in case No. 68, the patient died, not probably of acute rheumatism, but of typhus fever of a bad type, complicated with apthæ on the tongue and throat; besides, she only took the bark for four days, and had ceased to use it altogether for thirteen days before her death, not because it disagreed, but because it had no salutary effect.

In case No. 125, a suppression of urine was plainly the cause of the death. How far this disease might be connected with rheumatism seems very doubtful; the two disorders were probably independent of each other. The suppression of urine, there was reason to believe, had been brought on by habitual drunkenness with spirits. It is of importance to remark, that neither in this nor in any of the three other fatal cases, was there any inflammatory swelling of the joints when the bark was given; that it had receded in one, and had never appeared in the other two cases. Again, all these three patients were in such a state of extreme debility and languor, as to be apprehensive of sinking away into a syncope. With such symptoms, observes Dr. Haygarth, no candid physician would expect any mischievous effects from the bark.

In these circumstances it seemed preferable to all the remedies, although it had not sufficient power to save the patients' lives. According to the prevailing medical ideas on this subject, the greatest mischief should be apprehended from this remedy where the fever was high and the inflammation violent. On the whole, however, after the most rigid scrutiny, it seems manifest that not the slightest probability exists that the bark had disagreed or had aggravated a single symptom in any of the cases alluded to. It is very interesting and instructive to remark, that although the medicine here recommended was exhibited by Dr. Haygarth during the earliest stages of acute rheumatism, it seldom failed to produce very obvious effects in mitigating the symptoms; whereas in the few cases of dangerous complications, the worst symptoms were attributable obviously rather to such complications than to the remedy prescribed. The treatment which I now venture to recommend, observes Dr. Haygarth, is the result of gradual improvements for a long series of years, as successful experience led me by degrees to further deviation from the remedies usually employed in this disease. With sedulous attention to every circumstance which denotes that a medicine relieves or aggravates a patient's disorder, with a steady purpose to persevere in the former, and to avoid it in the latter circumstances, I have been long taught and thoroughly convinced that all hazard of doing mischief by means of it may be avoided, and yet that as much benefit may be obtained as its salutary qualities can produce.

By strictly following the rules and cautions dictated by the medical experience of others, and of my own, it has so happened that I never did witness, during a period of thirty years, a fatal consequence from any remedy which I had prescribed. So long and so uniform a course of successful experience has given much satisfaction to my own mind, and afforded a highly beneficial confidence in the safety of the practice; it banishes equally the injurious extremes of timidity and temerity; it encourages and warrants the efficacious method of treating diseases without rashness, or apprehensions of doing mischief.

Although directly contrary to the vulgar creed, and even to sceptical opinions maintained by men of knowledge in other sciences, there is no doubt that my accurate and faithful declaration of experience on the subject matter before us, confirmed by that of many other physicians of extensive practice, will have the eventual result of inducing others to adopt the same prectice. To those who may be strangers to the author's character, it may be necessary to remark, that he has never been guilty of any professional boastings or exaggerations. It cannot, therefore, be suspected that he should now make such a solemn and public asseveration, were it possible he could entertain the slightest doubt of the correctness of his statement. On a subject of such great importance, he feels it his duty to publish this general remark, conscious confidence of integrity places him above all personal considerations. To him, indeed, it seems most proper that a truth of so much importance should be adequately promulgated. It is not to be expected, however, that even this full and faithful statement of facts will immediately obtain general attention.

Though my respectable friend, Dr. Saunders, has recommended bark for rheumatism for many years, both in his lectures and publications, yet I do not know that it has anywhere received the attention which it justly merits; for even lately I have heard a pupil of Dr. Saunders censured by an intelligent and enlightened physician for prescribing it in this disease. For many years, whenever an opportunity presented itself, whether in correspondence, consultation, or even in casual conversation, I have for myself never ceased to recommend the Peruvian bark as incomparably the best remedy for the treatment of acute rheumatism.

When I first came to reside in London, I found the practice of prescribing bark in acute rheumatism, adopted by a very small number of the elderly physicians of the metropolis, and among these Dr. Saunders was the principal. It was adopted also, I have been credibly informed, by the then senior physicians of St. George's Hospital; but these must be considered as rare exceptions to the rule, for in a few years subsequently, I found my own representations of its value received with coolness, if not with distrust. The fact of its utter neglect in so short a time after its publication by so eminent a man as Dr. Haygarth, has always appeared to me an extraordinary phenomenon in morals. In Dr. Fothergill's time, there can be no doubt that it was frequently prescribed. Since that period, however, we have not often heard of it; and I have even known of public writers who, if I mistake not, have written strongly against its employment. "So loosely," observes one of these writers, "has the term rheumatism been applied, that a host of ailments with no character in common save that of pain have been classed under it, and much both of false experience and of bad practice has thence resulted. Diseases called rheumatic have been relieved by stimulant remedies, which, from the character thus acquired, have been empyrically resorted to in states of constitution for which they were utterly unsuited. In acute rheumatism, a disease intensely inflammatory, we have known the use of the most powerful stimulants confidently urged by well-meaning but misguided friends, who in support of their prescription have pleaded the wonderful cures which they had seen their favorite specific perform, in what they assumed to have been rheumatism: thus misled by a name, to recommend in active inflammation what could have benefitted only in a totally opposite state of the system. As many really rheumatic affections present an equivocal character, which to superficial observation too often appears to justify the use of stimulants, it is very necessary to discriminate the real disease, so as to distinguish it from those diseases with which it is liable to be confounded; and however difficult it may be to class the latter, it is better to leave their place in nosology unassigned, than by ranging them under the head of rheumatism, to beget confusion where clearness and precision are of the first importance." This reference, I presume, is intended to apply to Dr. Haygarth's practice, and to its adoption by a few competent practitioners who have seen it employed; if not, I am myself incompetent to guess at its intended application. If, on the contrary, I am correct in my supposition that the writer had the practice of Dr. Haygarth as the principal object of his remark, I am quite at a loss to account for his extreme arrogance, for who was Dr. Haygarth? He was confessedly one of the first physicians of his age, the friend and correspondent of Currie, Percival, Dobson, Clark, and many other professional writers of equal renown. He received the cinchona practice as delivered to him by Morton, Hulse, and Fothergill; continues it on a large scale of experience, without once feeling diffident of its value, and without incurring at least many examples of its failure for a period of five-and-thirty years, and who during the whole of that period attested its value by the strictest rules of induction. The reader is already acquainted with the manner in which I became impressed by Dr. Haygarth's practice. I beg now to inform him, that I have adopted it during the greater part of my professional life; and as it has seldom happened that I had ever occasion to prescribe it, except at an early period of acute rheumatism, I have great satisfaction in assuring the readers of the Lancet that I do not remember a case in which the disease was not happily subdued. I have often recommended it in cases of pure arthritic rheumatism during its acutest stage, and the disease has always yielded to the remedy; and I have also recommended it in violent pains of the joints, accompanied by alarming complications, but never in any one case injuriously to the interests of my patient. I have, therefore, no difficulty in recommending its adoption to my medical brethren, and especially to those who are most frequently favoured with the opportunities of seeing acute rheumatism in its earliest stages. From what I have already written upon the subject, I think it will be considered that its employment should, in every case, be anticipated by free abstraction of blood, as well as by the other important evacuations previously recommended to be resorted to. In a few cases it may be necessary to repeat such bleedings and other evacuations more than once; but I must own, that such a necessity has not often occurred within my experienoe. I feel myself entitled once more to repeat my confident assertion, that I consider the Peruvian bark the most powerful remedy that can be employed in an incipient case of acute rheumatism. Without exceptions in my practice, it has uniformly produced the most salutary effects. The pains, swellings, perspirations, and other symptoms of inflammatory fever, manifestly and speedily abate, and gradually cease, till health is perfectly restored. Before I conclude, I beg to repeat that the measure of bark which it has been my practice to recommend for the disease under consideration, has varied between a scruple and half a drachm, repeated three or four times daily.

The reader has already seen that the writers of the last and preceding centuries prescribed bark in much larger quantities. Dr. Haygarth states his practice to have been, p. 89 of his

"Clinical History," to order it in much more moderate doses. To sum up the whole in a few words, he observes, after the stomach and bowels have been sufficiently cleansed by antimony, I have, for many years, begun to order the Peruvian bark in doses of grs. x. or xv. every two, three, or four hours; and if this quantity has a salutary effect, it has been gradually increased to grs. xx., xxx., or xl., with sedulous attention never add more than what had perfectly agreed. It has generally been taken in milk, peppermint water, or the decoction of bark.

[*London Lancet.*

---

*Principal circumstances connected with seven cases, in which an Artificial Anus has been established in the adult for the relief of urgent symptoms produced by Stricture of the Rectum.* By W. H. Walshe, M. D.

[1. Fine (An. ?) *Vid Odier, Man. de Médecine*, p. 274, ed. 2, 1811.]—Female, æt. 70. Symptoms of gangrene from fæcal retention; tumour completely obliterating the rectum at its origin.

Opening made at "most prominent part of the abdomen;" one or two stools daily afterwards; patient lived more than a year, and died dropsical.

[2. Pillore (1776.) *Vid. L'Expérience, Janvier* 30, 1840.]—Adult male. No stool for upwards of a month; not a particle of two pounds of mercury, which had been exhibited by the mouth, discharged. Eight or nine inches of the end of the colon and beginning of the rectum totally obstructed from schirrhous induration, &c.

Transverse incision of the integuments above the fold of the right groin, and same of the cæcum; abundant fæcal evacuation. Peritonitic symptoms on the 20th; death on the 28th day. The bowels had not been completely emptied; the exact original weight of mercury being found in a knuckle of the jejunum, behind the bladder.

[3. Freer (1817.) *Vid. Med. and Phys. Journal*, vol. 45, p. 9, 1821.]—Male, æt. 47. Contraction of gut complete; attempt made to divide the stricture; no stool for ten days, vomiting, hiccup, &c.; "death inevitable."

Incision above and within anterior superior spine of the left ilium, and in descending colon; painful evacuation; patient, however, gradually sank, and died on the ninth day.

[4. Pring (1820.) *Vid. Med. and Phys. Journal*, vol. 45, p. 1, 1821.]—Widow, æt. 64. Obstruction seven inches above the anus; total retention of fæces for twelve days.

Incision within and above antero-superior spine of the left ilium, and in descending colon; immediate and forcible discharge of fæces. Three months after, indurated fæces passed through the natural anus, and continued to do so. Six months after, the patient was in good health.

[5. Martland (1824.) *Edinb. Med. and Surg. Journ.* p. 271, 1825.]—Male, æt. 44. A large tumour protruding, as it were, from the neck of the bladder; no stool for twenty-six days; marked stercoral tympanites.

Incision an inch above and within antero-superior spine of the left ilium, and in descending colon; instant escape of fæces and flatus; a year after the patient enjoyed good health; soft stools passed on a few occasions at one period through the natural anus.

[6. Amussat (1839). *Vid. Gazette Médicale, Oct.* 1839.]—Woman, æt. 48. No stool for upwards of *twenty-six* days; nausea, vomiting, &c. Hard, round, immoveable tumours, at the upper part of the rectum.

Incision in left lumbar region, and in descending colon. Four months after the operation, patient in excellent health: one or two evacuations daily; flatus passed by natural anus.

[7. Amussat (1839.) *Vid.* same paper.]—Case previously treated by breaking down fungous masses in the rectum, and subsequently by cauterization. Patient in a deplorable state.

Opening made in left lumbar region and in descending colon; no escape of fæces till the fifth day. Four months after, the patient in a better state than before the operation, and about to quit Paris for the country.

"When the disease is cancerous, the chances of ultimate advantage are, of course, vastly less than in cases of retention from simple induration; but even here it may be justifiably performed, provided the patient, after having been made fully acquainted with the nature and likelihood of the benefit to follow, still desires to undergo it." It is scarcely necessary to add, in defence of this position, that (even admitting the benefit obtained to be necessarily of short duration, which is by no means certain,) there are cases wherein the preservation of a given life, even for a few days, may be of the utmost consequence to families, in a worldly point of view.

It will be observed that in Pillore's case the unfortunate issue probably depended on the obstruction caused by the gravitation of the mercury into the pelvis: the site of the incision was also evidently ill-judged. The cause of death in Freer's case does not very clearly appear from his details.

M. Amussat's motives for preferring the operation proposed by Callisen (the lumbar incision, whereby implication of the peritoneum is avoided) to the inguino-abdominal incision of Littre, are explained in his papers referred to.

*Lond. Med. Gaz.*

